# Increased demand for amphetamine treatment in rural Australia

**DOI:** 10.1186/s13722-019-0144-6

**Published:** 2019-04-01

**Authors:** Julaine Allan, Ryan H. L. Ip, Michael Kemp, Nicole Snowdon

**Affiliations:** 1 Lives Lived Well, Orange, NSW Australia; 2 Conjoint Academic, National Drug and Alcohol Research Centre, UNSW, P.O. Box 9374, Orange East, NSW 2800 Australia; 3 Charles Sturt University, Wagga Wagga, NSW Australia; 4 Charles Sturt University, Orange, NSW Australia

**Keywords:** Methamphetamine, Amphetamine, Substance treatment, Rural Australia

## Abstract

**Background:**

A substantial increase in substance treatment episodes for methamphetamine problems suggests characteristics of the treatment population could have changed and that targeted treatment programs are required. To determine who methamphetamine treatment should be designed for this study has two aims. First, to empirically describe changes in amphetamine treatment presentations to a rural NSW drug and alcohol treatment agency over time. Second, to examine how these characteristics may affect the likelihood of being treated for amphetamines compared to other drugs.

**Method:**

The Australian Alcohol and Other Drug Treatment Services National Minimum Data Set (AODTS-NMDS) containing closed treatment episodes from a single agency from three time periods was used. Characteristics of people receiving amphetamine treatments in these three periods were compared and the effects of these characteristics on the odds of being treated for amphetamine were estimated using a logistic regression model. The characteristics utilised in the analysis include age, sex, Indigenous status, usual accommodation, living arrangement, source of referral and source of income.

**Results:**

The proportion of amphetamine treatment episodes doubled from 2006/2007 to 2015/2016 and overtook alcohol as the most commonly treated principal drug of concern. The estimated proportion of amphetamine treatments showed an increment across all ages and for men and women. It was found that younger people, women, people in temporary accommodation or homeless, people who were self-referred and people whose main source of income was not through employment are more likely to be treated for amphetamine use.

**Conclusion:**

Significant changes over time in the age, sex and Indigenous status of people receiving treatment for amphetamine as the principal drug of concern requires service delivery to match demand from younger people, particularly women; and Indigenous people. The needs and preferences for treatment of younger women who use amphetamine will be important factors in treatment planning service providers who are more used to providing treatment for young men who use cannabis and older men who use alcohol. Further research on women’s experiences in treatment and outcomes would be useful for informing treatment practices.

**Electronic supplementary material:**

The online version of this article (10.1186/s13722-019-0144-6) contains supplementary material, which is available to authorized users.

## Introduction

Australian media has reported an ice epidemic sweeping the country since the beginning of 2014. “Ice” is slang for crystal methamphetamine, a stimulant drug that is swallowed, smoked or injected. Methamphetamine use is not an epidemic in Australia. The population level use of methamphetamine has decreased from 2.1% in 2013 to 1.4% in 2016 [1]. However, those who use methamphetamine are using higher purity products more frequently and experiencing more negative consequences as a result [2]. Deaths from methamphetamine have doubled since 2009 and health impacts including heart problems and stroke have increased [3]. Significantly higher methamphetamine use is reported in rural and remote Australia compared to urban locations [4]. Hereafter the term used will be amphetamine as it is commonly referred to in Australian government reports and surveys [1, 5].

There is an increasing need for health services to engage with people who use amphetamines [6], and new harm minimisation practices and targeted treatment programs are required relevant to the context of amphetamine users experience [5]. Context is a critical factor in healthcare delivery yet is typically under-estimated when planning services [7]. Ideally, targeted treatment and harm minimisation practices are context specific. That is, they should be located close to areas of highest need, acceptable to the age, sex and cultural background of potential clients; and deliver a comprehensive service that does not isolate drug use from a person’s other concerns or needs [8, 9]. For rural populations, interventions should be specific to the unique circumstances of rural settings [4].

The characteristics of those the program or treatment is designed for is one of the key domains in program implementation [10]. The characteristics of amphetamine users will be important to responding effectively from existing treatment services. Detailed examination of who is accessing treatment services is required to define who amphetamine treatment should be designed for.

The Australian National Drug and Alcohol Treatment Data Report provides a broad overview of treatment provided and drills down to some demographic and drug specific factors [11]. However, it does not consider multiple variables at a time nor provide enough detail for a localised approach to substance treatment planning.

An in-depth examination of a single agency is one way of contextualising demand for, and supply of substance treatment services to appropriately inform service delivery. Systematically analysing and reporting local data can determine which user factors need to be responded to. Examining changes over time can shape service provision in response to changing demand. This study had two aims. First, to empirically describe changes in amphetamine treatment presentations to a rural NSW drug and alcohol treatment agency over time. Second, to examine how these characteristics may affect the likelihood of being treated for amphetamines compared to other drugs.

## Study context

Australian drug and alcohol treatment programs are provided by both government and non-government agencies. The Australian Alcohol and Other Drug Treatment Services National Minimum Data Set (AODTS-NMDS) measures supply of publicly funded mainstream drug and alcohol treatment. AODTS-NMDS data is collected by individual agencies and reported to state and territory health authorities [11]. In New South Wales (NSW) one-third of service providers are non-government agencies who provide about half of all treatment episodes in the state [11].

Lives Lived Well—NSW (LLW) is a non-government drug and alcohol treatment service based in the regional NSW town of Orange (2016 population 39,755). Orange is a regional centre for agricultural industries including apples and wine grapes; gold mining and health care facilities including the largest psychiatric hospital in NSW. LLW—NSW was established in 1980 with a residential drug and alcohol rehabilitation centre and had expanded by 2004 to also have assessment, residential withdrawal, medical and outpatient counselling and case management services. LLW—NSW provided around 2400 treatment episodes annually across the various treatment types to men (65% of treatment episodes) and women (35%). Two-thirds of people who accessed LLW services were from western NSW; and one-third came from cities on the coast—usually to inpatient programs. All LLW services were government funded from either state or commonwealth health departments. Outpatient services were free, inpatient withdrawal cost $120 AUD per admission and the 90-day residential rehabilitation program cost participants $120 AUD per week. Between 2006 and 2016 the withdrawal fees doubled but the rehabilitation fees were reduced by $20 per week as government welfare payments decreased and became harder to obtain.

National and state governments both provided new funding for amphetamine treatment following a series of Ice taskforces and Parliamentary Inquiries into the prevalence of and harms related to amphetamine use. However, it was 2017 before new services or service enhancements were funded and they were not considered a factor in influencing demand in this study. Prior to 2017 none of the government funding specified treatment for a type of drug or for a particular group of people other than those over eighteen. Services were expected to be responsive to demand and provide services consistent with the substance treatment evidence base.

Treatment provided by the agency included individual and group counselling using Cognitive Behavioural Therapy and Motivational Interviewing approaches. Psycho-educational groups included relapse prevention strategies, sexual health, legal advice and financial counselling. The inpatient withdrawal program had medical staff who conducted health checks as well as provided medication for withdrawal management. The residential rehabilitation program also included cognitive remediation programs for people with cognitive impairments; a health and fitness program at a local gym; and arts and crafts options including music lessons and drumming. Rehabilitation residents were invited to attend the local Alcoholics Anonymous group but meetings were not compulsory.

Prior analyses of LLWs treatment episode data have identified alcohol (60% of episodes), opioids (18%) and cannabis (18%) as the three most common drugs treatment was provided for [12]. The average age of people accessing services was thirty-seven. In 2015 LLW staff reported increased demand for treatment from people who were using amphetamines. People using amphetamines were perceived by staff as having different needs for treatment compared to those primarily using depressants and less likely to complete treatment than people using other drugs. The perceived differences had not been explored. Around the same time researchers were arguing against an ‘ice epidemic’ and pointing to unchanged population data on drug use that refuted the increased harms treatment services were seeing [5, 13].

Lives Lived Well’s research program has used both state-wide and local treatment episode data to highlight the need for treatment services to address homelessness in treatment planning [12]; identify the prevalence of people in treatment who have a cognitive impairment [14] and investigate access of Aboriginal women to drug and alcohol treatment [15]. To appropriately shape service delivery an analysis of amphetamine treatment episodes was required.

## Method

The AODTS-NMDS containing closed treatment episodes from LLW from July 2006 to June 2007, July 2010 to June 2011, and January 2015 to June 2016 were used. A ten-year period was selected for analysis that commenced when a consistent data collection method that included checking data quality was implemented. Further, increased amphetamine use was reported in 2006 including the first media references to an ‘ice epidemic’ and related government policy responses [16].

The focus was on the principal drug of concern and the samples were split into episodes where the principal drug of concern was amphetamine and episodes where the principal drug of concern included all other drugs. Characteristics, including age, sex, Indigenous status, usual accommodation, living arrangement, source of referral, and source of income were extracted from the datasets. These characteristics among amphetamine treatments over the three time periods were compared using the Marascuilo’s procedure [17].

The effects of the above characteristics on the odds of being treated for amphetamine were assessed using a logistic regression model with the three datasets combined. Covariates included the above characteristics, time period, and the first-order interaction between each pair of time period, age and sex. The logarithm of the odds ratio of being treated for amphetamine against other drugs was used as the response variable.

For some variables there were several categories that occurred rarely in AODTS-NMDS. These categories were therefore combined or moved to the “Others” category (see Additional File 1). The categories were grouped in this way for the ease of interpretation and to avoid having too few episodes in some categories. Episodes with age below 15 years or above 60 years were removed due to the low number of entries for these age groups. For each qualitative variable, the level that occurred the most frequently in the 2015/2016 period was used as the baseline level in the logistic regression model.

Specifically, the logistic regression model uses

$${ \log }\left( {{\text{odds}}\left( {Amphetamine} \right)} \right) = \log \left[ {\frac{{P\left( {Amphetamine} \right)}}{{1 - P\left( {Amphetamine} \right)}}} \right] = \beta_{0} + \varvec{\beta^{\prime}x},$$

where $\beta_{0}$ is the intercept term, $\varvec{\beta}$ is the vector of coefficients and $\varvec{x}$ is the vector of covariates. For covariates that do not interact with other variables, the estimated odds ratios were calculated as $\exp \left( {\hat{\beta }} \right)$, where $\hat{\beta }$ is the estimated coefficient of a variable. When the covariates were fixed, the proportion of amphetamine treatment episodes can be estimated as

$$\hat{P}\left( {Amphetamine} \right) = \frac{{\exp \left( {\hat{\beta }_{0} + \hat{\varvec{\beta }}^{\prime } \varvec{x}} \right)}}{{1 + \exp \left( {\hat{\beta }_{0} + \hat{\varvec{\beta }}^{\prime } \varvec{x}} \right)}}.$$

In all analyses, the level of significance was set at 5%. The analyses were carried out using R [18].

People’s names were removed prior to LLW supplying the data. Ethical approval to use de-identified data was granted by Charles Sturt University’s Human Research Ethics Committee (Protocol No. 2013*/*016).

## Results

There were 2806, 3435 and 2681 closed treatment episodes in the three time periods, respectively. Among these episodes, amphetamine was the principal drug of concern in 412 (14.7%), 257 (7.5%) and 904 (33.7%) of the episodes in the datasets, respectively. This is a similar trend to the national treatment data of 12%, 9% and 23% during the respective time periods [11] while reported use in the Australian population has continued to fall from 2.5% in 2007 to 1.4% in 2016 [1]. However, a significantly higher percentage of episodes were for amphetamine treatment in the LLW 2015-16 dataset than were recorded nationally (Z = 13.8, p < 0.001). Treatment episode data included an ‘other drug of concern’ category. Amphetamine decreased as a secondary drug over the study period. In 2006/2007, 470 people (20%) had amphetamine identified as a secondary drug of concern. Whereas 472 people (15%) and 218 people (12%) identified amphetamine in 2010/2011 and 2015/2016, respectively.

**Fig. 1:**
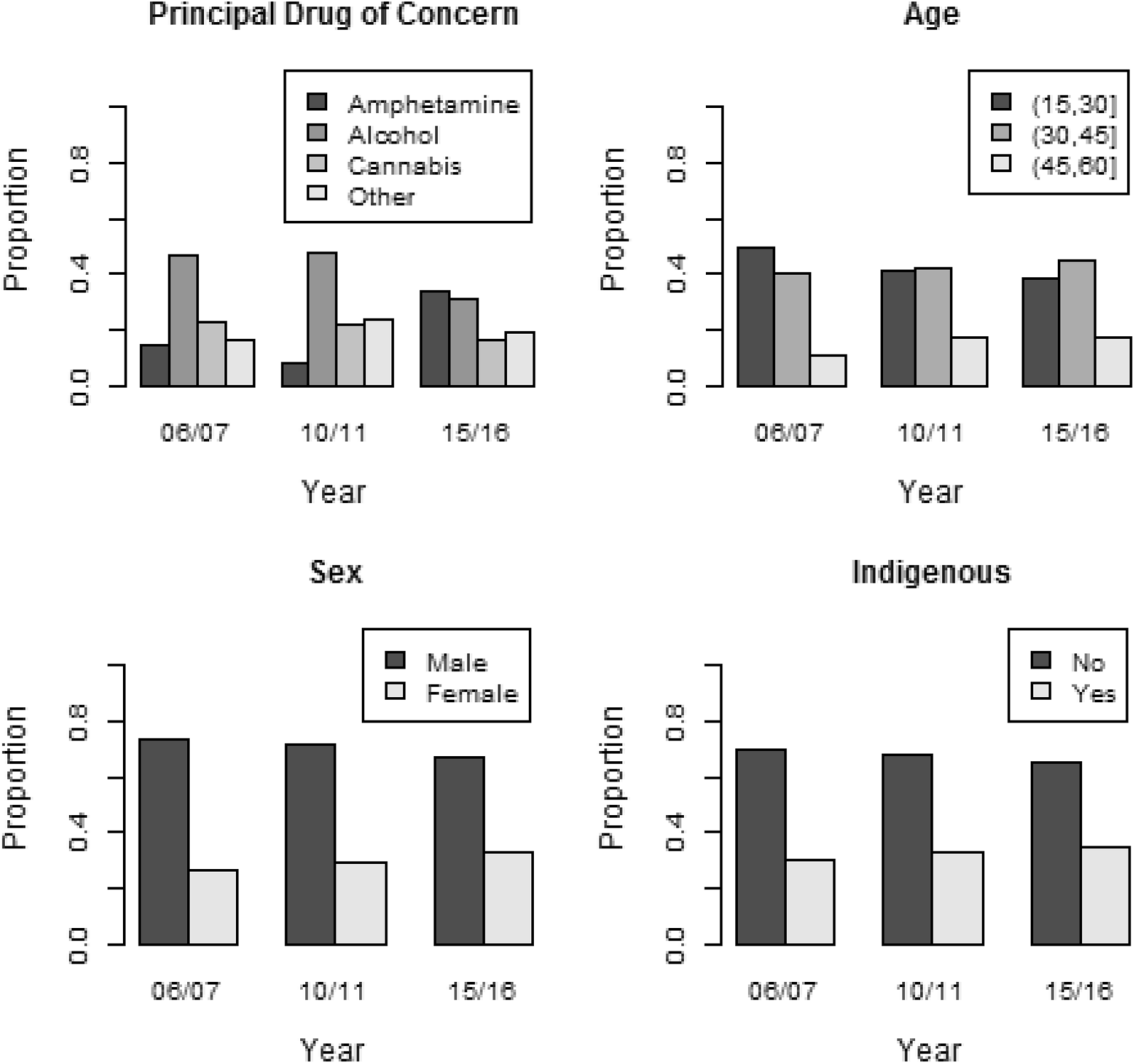
Snapshots of LLW client profiles in the three periods: principal drug of concern (top-left), sex (top-right), age (bottom-left), and Indigenous status (bottom-right)

Figure 1 shows the client profile of LLW according to the principal drug of concern, sex, age and Indigenous status. In 2015/2016, amphetamine overtook alcohol as the most common principal drug of concern. The proportions of both female and Indigenous episodes increased over time. The proportion of episodes for people below 30 years of age decreased overall.

**Fig. 2:**
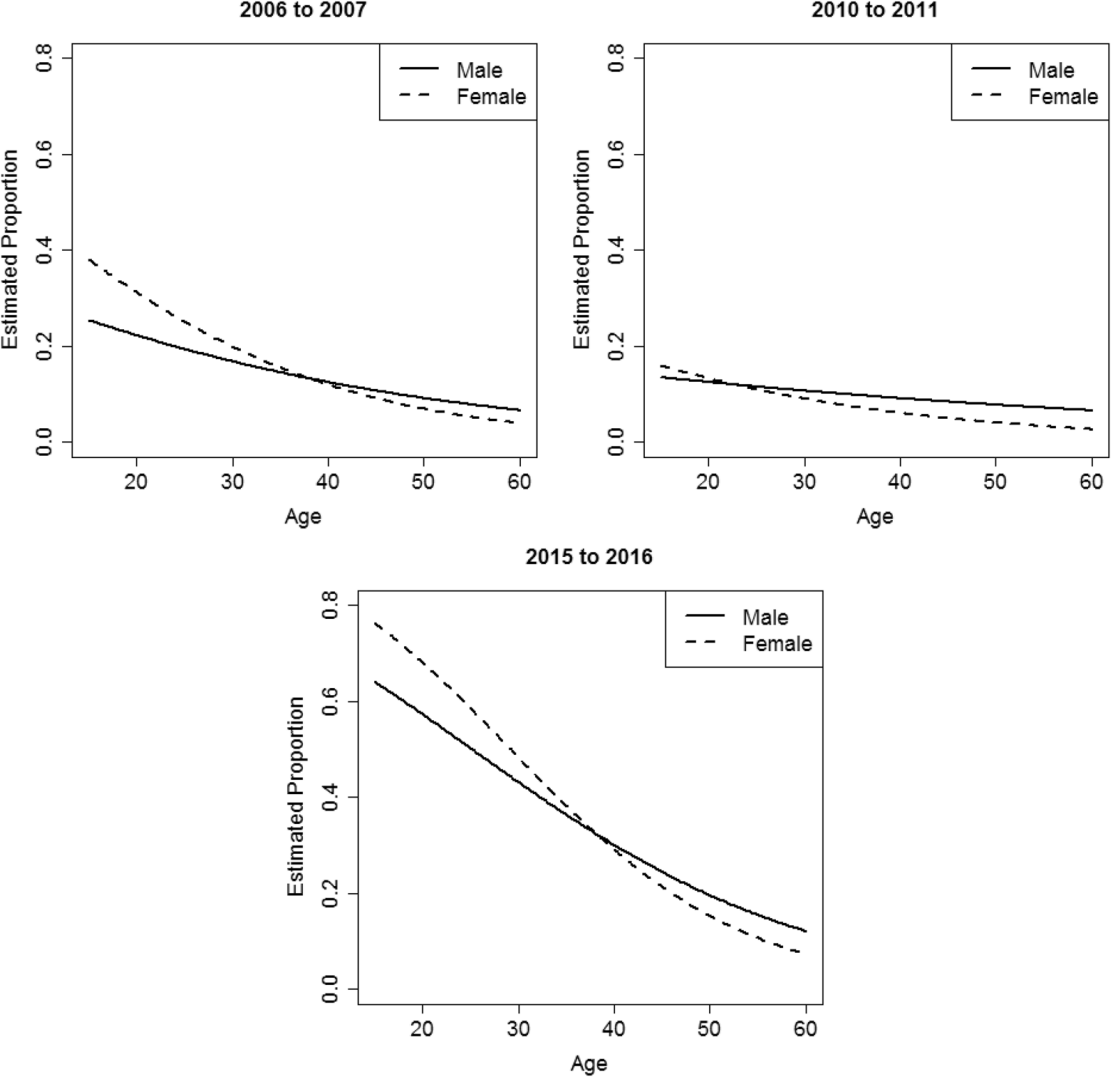
Estimated proportion of amphetamine treatment episodes for clients with baseline characteristics by sex and age in 2006/2007 (top-left), 2010/2011 (top-right) and 2015/2016 (bottom)

Table 1 shows the characteristics of clients whose principal drug of concern was amphetamine in the three time periods. Figure 2 shows the proportion of amphetamine episodes by sex and age in different periods estimated from the logistic regression model. Since these three variables interact with each other, the marginal effect of each variable depends on levels of the other variables. The estimated coefficients and the variance–covariance matrix of the coefficients are provided in Additional files 2 and 3. This would allow computation of the marginal and interacting effects, following the method described in Norton et al. [19] and Karaca-Mandic et al. [20].

**Table 1:** Characteristics of clients whose principal drug of concern was amphetamine

Period	2006/2007	2010/2011	2015/2016
Number of episodes	412	257	904
*Sex*			
Male (%)	69.7a	74.7b	64.8a
Female (%)	30.3b	25.3a	35.2b
*Age*			
> 15 and ≤ 30 (%)	63.3b	46.3a	49.7a
> 30 and ≤ 45 (%)	35.0a	48.6a	46.2a
> 45 and ≤ 60 (%)	1.7a	5.1ab	4.1b
*Indigenous*			
Yes (%)	29.4a	26.5a	40.0b
No (%)	68.9b	72.4b	60.0a
*Living arrangement*			
Alone (%)	32.5	25.3	28.2
With parents or friends or relatives (%)	38.3a	49.4b	43.4b
With spouse/partner and/or child(ren) (%)	23.8	24.1	18.0
Unknown/others (%)	5.3b	1.2a	10.4c
*Usual accommodation*			
Owned or rented (%)	77.9a	89.1b	79.3a
Temporary accommodation or homeless (%)	20.9b	10.1a	11.2a
Unknown/others (%)	1.2a	0.8a	9.5b
*Source of referral*			
Self (%)	30.1a	42.8b	50.8b
Legal (%)	24.3	24.9	22.8
Health care (%)	13.6b	14.8b	6.6a
Other AOD (%)	19.4b	10.9a	10.3a
Others (%)	12.6b	6.6a	9.5ab
*Source of income*			
Not through employment (%)	91.7b	89.1ab	84.6a
Through employment (%)	6.1	6.6	3.4
No income (%)	1.0a	3.9a	4.8b
Unknown (%)	1.2a	0.4a	7.2b

Significant differences in percentages within a row based on Marascuilo’s multiple comparison at a family-wise error rate of 5% are indicated by different alphabetical letters. The percentages may not sum up to 100 due to either rounding or missing values

Table 2 shows the effects of the non-interacting variables on the odds of being treated for amphetamine estimated from the logistic regression models. The reference levels of qualitative variables define the “baseline characteristics” for a treatment episode as a non-Indigenous male who owned or rented a place for living, who lived with parents, friends or relatives, self-referred to the agency, and whose main source of income was not through employment.

**Table 2:** Effect estimates for non-interacting variables from the logistic regression model

	Odds Ratio	95% Wald CI	*p* value
*Indigenous*			
Yes	0.920	(0.808, 1.047)	0.207
No	Ref	–	–
*Living arrangement*			
Alone	0.923	(0.785, 1.085)	0.330
With spouse/partner and/or child(ren)	1.000	(0.850, 1.176)	0.999
Unknown/others	0.892	(0.628, 1.267)	0.522
With parents or friends or relatives	Ref	–	–
*Usual accommodation*			
Temporary accommodation or homeless	1.815	(1.473, 2.237)	< 0.001
Unknown/others	0.683	(0.464, 1.006)	0.053
Owned or rented	Ref	–	–
*Source of referral*			
Legal	0.859	(0.734, 1.005)	0.057
Health care	0.777	(0.632, 0.955)	0.017
Other AOD	1.067	(0.879, 1.294)	0.512
Others	0.806	(0.652, 0.996)	0.046
Self	Ref	–	–
*Source of income*			
Through employment	0.357	(0.276, 0.463)	< 0.001
No income	1.128	(0.801, 1.589)	0.490
Unknown	0.513	(0.372, 0.709)	< 0.001
Not through employment	Ref	–	–

Residual deviance: 7073.1 on 8778 *df*. See also Additional file 2 for coefficient estimates for variables that interact with other variables. The *p* values were calculated based on *z* test

### Age and sex

The proportion of amphetamine treatment episodes in 2015/2016 was estimated to be the highest across all ages for both sexes (Fig. 2). Meanwhile, in all periods, episodes for younger people were more likely to have amphetamine as the principal drug of concern, rather than other drugs (Fig. 2). In 2015/2016, the odds of having amphetamine as the principal drug of concern was estimated to reduce by around 5% and 8% for every year increase in age for males and females, respectively. For episodes provided to people under 40 years of age, there was a higher proportion of female clients with amphetamine as the principal drug of concern, compared to males. The trend reversed for episodes for people above 40 years of age (Fig. 2). The estimated proportion between male and female changed around age 25 in 2010/2011. This was different from the change that occurred around age 40 in both 2006/2007 and 2015/2016 (Fig. 2). While our model estimated that most younger females were primarily treated for amphetamine in 2015/2016 (Fig. 2), most of the treatment services were provided to males (Table 1).

### Indigenous status

The proportion of Indigenous people among amphetamine treatment episodes increased from around 30 to 40% over the 10-year period used in the analysis (Table 1). Indigenous people in treatment were likely to be younger and female compared to non-Indigenous people. There are no significant differences between Indigenous and non-Indigenous people in terms of the odds of being treated for amphetamine, after considering all other variables (Table 2).

### Living arrangements and usual accommodation

Among the amphetamine treatments, there was a significant increase in the proportion of episodes for people living with parents, friends or relatives in 2010/2011 and 2015/2016, compared to 2006/2007 (Table 1). However, the logistic regression model showed that these living arrangements did not result in significantly different odds of being treated for amphetamine (Table 2).

Homeless people or those in temporary accommodation significantly reduced from around 20% in 2006/2007 to around 10% in the following two periods (Table 1). However, the logistic regression model demonstrates that despite the reduction in overall numbers homeless people or people in temporary accommodation are 1.8 times more likely to have amphetamine as their principal drug of concern compared to those owning or renting accommodation (Table 2).

### Referral source

Among the amphetamine treatment episodes, most of the clients were self-referred. The proportion of self-referrals significantly increased from around 30% in 2006/2007 to around 50% of amphetamine treatment episodes in 2015/2016. Within the same period, the proportion of episodes referred from health care settings and other AOD significantly dropped (Table 1). Compared to those who were self-referred, people who were referred from health care settings were significantly less likely to be treated for amphetamine (Table 2).

### Source of income

Among the amphetamine treatment episodes, most people had income through sources other than employment. Although the proportion dropped significantly from 92 to 85% from 2006/2007 to 2015/2016, the proportion of clients who did not have any income at all increased significantly from 1 to 5% in the same period. Table 2 shows that, the odds of being treated for amphetamine for people with income through employment was 0.38 times that for clients whose incomes were not through employment.

## Discussion

Both the number and proportion of treatment episodes provided for amphetamine in the organisation studied has substantially increased over time. The proportion of treatment episodes provided for amphetamine was significantly greater than the percentage reported nationally [11]. There were changes over time in the age, sex and Indigenous status of people receiving treatment for amphetamine as the principal drug of concern. It is estimated that more than half of treatment episodes provided for people aged 30 or below were for amphetamine. Alcohol was no longer the principal drug of concern for most treatment episodes. The reduction in amphetamine as a secondary drug of concern at the same time as it increased as a principle drug supports the idea that people were experiencing more harms from methamphetamine as it became stronger and it became the main drug causing them problems. Many people in treatment are poly drug users and drug use patterns change according to what is available at the time.

The biggest change is in the proportions of younger women represented in the treatment episodes. While treatment episodes for amphetamine use have typically been provided to men younger than thirty-five there is now a high proportion of women in that group that exceeds the proportion of amphetamine treatment episodes provided to men. Women usually comprise around one-third of the treatment population overall and treatment has been designed for the needs and preferences of men, particularly in residential treatment [21, 22]. For example, in the LLW residential units, vocational activities have a construction, mechanical and labouring focus; recreational activities are held in a weight lifting gym; and there are no facilities for children’s activities during weekend visits. In the treatment groups, relapse prevention activities and role play primarily examine risks and triggers likely in pubs and sporting events while promoting the role of female family members in supporting sobriety. The analysis and subsequent examination of psychosocial treatment activities has revealed the strongly gendered assumptions of the treatment activities. The needs and preferences for treatment of younger women who use amphetamines will be important factors in changing treatment for service providers who were used to providing treatment for young men who use cannabis and older men who use alcohol.

The nexus between child protection, criminal justice, domestic violence, child and family services and drug and alcohol problems is well documented [21, 8]. Younger women and men represented in the data used in this study are likely to have children in their care and/or be involved with child protection agencies who will be monitoring the outcome of any treatment programs. With more younger women in treatment programs, understanding women’s experiences and outcomes from different treatment types would be useful for directing women into the most appropriate and effective service types rather than assessing them on drug use alone. Child protection agencies may be a factor in increased amphetamine treatment episodes as women in particular are required to attend treatment to retain custody of their children.

Younger people in treatment services are likely to have different interests, concerns and healthcare needs compared to older people who are dependent on alcohol. Physical problems and capacity to recover from illness and injury are highly likely to be different with younger people less likely to have chronic illnesses. Homelessness, temporary or unstable accommodation, involvement with the legal sector and unemployment indicates many people in drug and alcohol treatment for amphetamine use will need a range of supports to change their current circumstances [10, 13]. Substance treatment services will have to work in a collaborative and coordinated way with agencies and services outside the healthcare sector to improve and sustain treatment outcomes for people. Self -referral is the most common pathway into treatment services and few people are referred from other health services. This suggests a lack of coordination and cooperation between health and community sector agencies. LLW’s ideal workforce will be one that is experienced and knowledgeable in cross sector work as well as capable and permitted to respond to the situational factors people in substance treatment are facing.

Capability to offer a culturally contextualised service is critical for Lives Lived Well. The number of Indigenous people represented in this study’s treatment episodes is a factor of location in regional NSW where the Indigenous population is one of the highest in Australia but also because Indigenous people are overrepresented in problem drug use statistics nationally [23, 11]. Strategies such as ensuring there are a proportion of Indigenous staff to match the number of clients and involving Elders in services and in consumer representation groups will be important to ensure that localised cultural practices are embedded within service delivery [15, 24].

The age and sex profile of alcohol treatment episodes is different to those of amphetamine episodes. While alcohol use has declined in Australia overall it is still the drug that causes the most harm after tobacco [11]. The proportion of episodes provided for alcohol by LLW has declined and the agency should consider if alcohol dependent people are unable to access treatment because of the number of amphetamine treatment episodes being supplied. The public focus on amphetamine use may have caused an increase in amphetamine treatment episodes. The Australian government Ice Taskforce investigations and reports generated several national health promotion campaigns about harms associated with methamphetamine use. The campaigns included television advertising depicting amphetamine users being violent to others. These campaigns alongside the media reports of an ‘ice epidemic’ may have resulted in more people seeking treatment in 2015/2016 because they were made aware of both harms and treatment options. Qualitative interviews with amphetamine users and staff of treatment centres could explore what drives trends in treatment episodes and how changes are responded to. This is an important area of research for the future.

## Limitations

The study reports the results from an observational study that represents observed patterns in reported treatment episodes only. Any relationships reported should not be considered as causal relationships. Further, the recorded treatment episodes may include the same individual many times within the same and different treatment types. While this suggests that amphetamine users may access treatment more often it cannot be confirmed from the existing data. Therefore, the results should be interpreted with caution. Analyses using identified data and statistical linkage keys would assist with reporting treatment usage and access patterns.

There are some questions arising from the study results that require further investigation. People come to the LLW rural services from all over New South Wales with more than half usually residing in rural or regional areas. There are consistent reports of higher methamphetamine use in rural areas [4]. The differences in treatment episodes between rural and urban dwellers were not examined in this study are important to include in future work. The age differences shown in Fig. 2 when the estimated proportion of amphetamine treatment episodes between male and female in 2010/2011 changed around age 25 instead of age 40 in the other datasets is unexplained. Further analysis over time could identify if this was an anomaly or something that recurs.

## Conclusion

Together with an increased number of amphetamine treatment episodes there were several changes in the characteristics of people using LLW treatment services. LLW must assess their treatment types to ensure they are contextualising service delivery to demand from younger people, particularly the needs of young women; are providing appropriate support for Indigenous people and addressing homelessness. Increased amphetamine treatment episodes have been noted nationally. It is likely similar changes in the treatment population will be experienced in other agencies. All treatment services should review who demand is coming from and what is supplied to ensure the best treatment options are provided.

## Additional files

**Additional file 1**: Variable Grouping.

**Additional file 2**: Estimated coefficients (slopes) for the logistic regression model.

**Additional file 3**: Estimated variance-covariance matrix for the estimated parameters in the logistic regression model.

## Abbreviations

ABS: Australian Bureau of Statistics
AIHW: Australian Institute of Health and Welfare
AODTS-NMDS: The Australian Alcohol and Other Drug Treatment Services National Minimum Data Set
LLW: Lives Lived Well
NSW: New South Wales
