# Supplementary material for: Increased demand for amphetamine treatment in rural Australia
Source: Addict Sci Clin Pract. 2019 Apr 1;14:13. doi: 10.1186/s13722-019-0144-6 (PMC6442410; doi:10.1186/s13722-019-0144-6)
Supplement: Supplementary file 1 — Additional file 1: Variable Grouping. [file 13722_2019_144_MOESM1_ESM.pdf]

Additional File 1: Variable Grouping

Variable	Original categories in AODTS-NMDS	Combined to
Sex	Male	Male
	Female	Female
	Not stated	Removed
Indigenous status	Aboriginal but not Torres Strait Islander origin, Torres Strait Islander but not Aboriginal origin, Aboriginal and Torres Strait Islander origin	Indigenous (Yes)
	Neither Aboriginal nor Torres Strait Islander origin, Not stated	Non- Indigenous (No)
Living arrangement	Alone	Alone
	Parent(s), Other relative(s), Friend(s), Friend(s)/parent(s)/relative(s)	With parents or friends or relatives
	Spouse/partner, Alone with child(ren), Spouse/partner and child(ren)	With spouse/ partner and/ or child(ren)
	Other, Not known/not stated	Unknown/ Others
Usual accommodation type	Rented house or flat (public or private), Privately owned house or flat	Owned or rented
	Boarding house, Hostel/supported accommodation services, Psychiatric hospital, Alcohol/other drug treatment residence, Shelter/refuge, Prison/detention centre, Caravan on a serviced site, No usual residence/homeless	Temporary accommodation or homeless
	Other, Not known	Unknown/ Others
Referral source	Self	Self
	Police diversion, Court diversion, Other criminal justice setting, Family and child protection service	Legal setting
	General practitioner, Medical officer/specialist, Psychiatric hospital, Other hospital, Residential community mental health care unit, Other residential community care unit, Non-	Health care setting

	residential community mental health centre, Non-residential community health centre	
	Residential alcohol and other drug treatment agency, Non-residential alcohol and other drug treatment agency	Other AOD
	Family member/friend, Workplace, Other, Not stated	Others
Source of income	Temporary benefit, Pension, Student allowance, Dependent on others, Retirement fund	Not through employment
	Full-time employment, Part-time employment	Through employment
	No income	No income
	Other, Not stated/not known	Unknown
